# Assessment of Disease-related Knowledge Among Children With Inflammatory Bowel Disease and their Family Using IBD-KID2: Evaluating Tool Generalizability

**DOI:** 10.1097/PG9.0000000000000093

**Published:** 2021-07-12

**Authors:** Angharad Vernon-Roberts, Robert N. Lopez, Peter Lewindon, Daniel A. Lemberg, Nerissa L. Bowcock, George Alex, Anthony Otley, Kevan Jacobson, Amin J. Roberts, Helen M. Evans, Richard B. Gearry, Andrew S. Day

**Affiliations:** From the * Department of Paediatrics, University of Otago (Christchurch), Christchurch, New Zealand; † Department of Paediatric Gastroenterology, Queensland Children’s Hospital, Brisbane, Australia; ‡ Department of Paediatric Gastroenterology, Sydney Children’s Hospital, Australia; § Department of Paediatric Gastroenterology, School of Women’s and Children’s Health, University of New South Wales, Sydney, Australia; ∥ Department of Paediatric Gastroenterology, Royal Children’s Hospital, Melbourne, Australia; ¶ Department of Pediatrics, Dalhousie University, Nova Scotia, Canada; # Department of Paediatric Gastroenterology, BC Children’s Hospital, Vancouver, British Columbia, Canada; ** Department of Paediatric Gastroenterology, Starship Child Health, Auckland, New Zealand; †† Department of Medicine, University of Otago (Christchurch), Christchurch, New Zealand.

**Keywords:** external validity, parents, siblings, score correlation

## Abstract

Children with inflammatory bowel disease (IBD) and their families benefit from improved knowledge of their disease and treatment. Knowledge levels of individual family members are infrequently studied but may identify where education is best directed. We aimed to assess disease-specific knowledge among children with IBD, parents, and siblings, using a validated assessment tool (IBD-KID2), and to establish generalizability of IBD-KID2.

**Methods:**

Children with IBD and family members were recruited from tertiary IBD clinics in New Zealand, Australia, and Canada. All participants completed IBD-KID2 online at baseline, and the children with IBD again after 2 weeks to assess reliability.

**Results:**

Participants included 130 children with IBD, 118 mothers, 55 fathers, and 37 siblings. Children with IBD had a mean score of 9.1 (SD 2.9) (maximum 15 points), significantly lower than parents (*P* < 0.005) and higher than siblings (*P* < 0.005). Scores of children with IBD were positively associated with current age (*P* < 0.005), age at diagnosis (*P* = 0.04) and fathers education level (*P* = 0.02). Significant score correlations were seen between children with IBD and their mother (*P* < 0.005) but not father. Sibling scores were not correlated with either parent. Test–retest reliability was high. The cohorts from each country were comparable, and no difference in group scores was seen between countries.

**Conclusion:**

IBD-KID2 is a generalizable and reliable tool for the assessment of disease and treatment knowledge for children with IBD and their families. Score correlations between parents and children with IBD suggest transfer of knowledge, but sibling knowledge is low and targeted education may be beneficial.

## Abstract 2

Supplemental Digital Content is available in the text.

**What Is Known**

- IBD-KID2 is a valid and reliable tool for the assessment of disease-specific knowledge for children with IBD.
- The provision of disease and treatment information, as well as good parent-sibling communication about illness, is a protective factor against sibling psychosocial issues.
- Little is known of disease-specific IBD knowledge among the family unit as a whole, in particular, the siblings of children with IBD.

**What Is New**

- IBD-KID2 is generalizable to the wider population of children with IBD.
- Siblings have poor disease-specific knowledge of IBD and may benefit from targeted education.

## INTRODUCTION

When a child is diagnosed with a chronic condition such as inflammatory bowel disease (IBD), the whole family unit must adjust to the burden of disease, its management, and the emotional and social consequences that this may bring (1–3). For children with IBD, their treatment adherence and the development of self-management skills may benefit from addressing modifiable factors such as disease and treatment knowledge (4,5). For siblings of children with chronic illnesses, their psychosocial outcomes are improved by greater disease-specific knowledge (1,3,6,7).

The assessment of disease-specific knowledge among children with IBD helps target education in those areas where gaps or misunderstandings lie, and family members should also be considered to benefit from such input. While the knowledge levels of children with IBD and their parents have been studied previously (8–10), little attention has been given to studying siblings or the family unit as a whole. The concurrent assessment of knowledge levels for all family members may establish whether parent knowledge is associated with that of their children, thereby highlighting where the greatest impact of education strategies may lie; in teaching the parents so they can impart information, or to additionally target education at siblings to directly enhance their understanding and psychosocial outcomes.

The aims of this study were 2-fold; to use a validated IBD knowledge assessment tool, IBD-KID2 (11), and to measure the knowledge levels of children with IBD and their family members. While IBD-KID2 has previously been shown to have validity and reliability, external validity (generalizability), has not yet been established outside the center where it was developed, thereby limiting the extent to which the original findings can be extrapolated to other IBD cohorts in other centers and countries. This study, therefore, also aimed to establish external validity by measuring IBD knowledge levels among families in a number of geographic locations where English is the common language.

## METHODS

### Collaborating Centers and Ethics

This study was performed as a multicenter collaboration in a number of tertiary care hospitals in 3 countries, chosen for their geographic diversity and having English as the first language spoken in their region. Ethics and Research Governance approvals were obtained from all collaborating centers, with individual center approval numbers provided in brackets.

*New Zealand*: Christchurch Hospital, Christchurch (H16/116), Starship Children’s Hospital, Auckland (A+8353).

*Australia*: Multicenter site approval was granted via the Australian National Mutual Acceptance Scheme (HRC/18/SCHN/432), and individual applications made for Site-Specific Research Governance approval: Sydney Children’s Hospital, Sydney (SSA/18/SCHN/465), Queensland Children’s Hospital, Brisbane (SSA/18/QCHQ/47719), Royal Children’s Hospital, Melbourne (SSA/47719/RCHM-2018-157604).

*Canada*: IWK Health Centre, Nova Scotia (1023997), BC Children’s Hospital, Vancouver (H18-02697).

### Population and Setting

Children with IBD and their families were approached during IBD outpatient clinics at each center. Inclusion criteria for the study were as follows: children with IBD of age 8 years and over, at least 1 parent for every child with IBD, any siblings of age 8 years and over who wished to participate. There were no exclusion criteria.

### Study Process

The consent process was determined by the Research Ethics Board stipulations regarding data sharing at each collaborating center, but all participants completed a signed consent or assent either on paper or digitally. IBD-KID2 assessments were completed electronically by participants using a HIPAA compliant online form provider (Cognito Forms). To establish test–retest reliability of IBD-KID2 in the target population, children with IBD were asked to complete IBD-KID2 twice, with the repeat assessment sent 2 weeks after the initial assessment.

Demographic data were collected from all participants regarding their age and gender. Additionally, siblings were asked if they had IBD. Individual information on their diagnosis was collected from the children with IBD, and parents were asked for their highest level of education achieved, whether they also had IBD, and whether they belong to an IBD support group. Ethnicity data were not collected due to the different nationalities and populations in each country.

### IBD-KID2

The sole outcome measure of the study was the IBD-KID2 knowledge assessment survey (11) (Appendix 1, Supplemental Digital Content *http://links.lww.com/PG9/A49*). IBD-KID2 takes the format of 15 questions: 9 true/false and 6 multiple choices. Each question also includes a “don’t know” response option. Participant responses are scored as 1 for each correct answer, to a maximum total of 15. Knowledge domains included general IBD, treatment, lifestyle, and nutrition.

### Statistical Analysis

Mean IBD-KID2 scores were calculated for each participant group: children with IBD, mothers, fathers, and siblings, and compared using analysis of variance (ANOVA) with post-hoc analysis using Tukey’s test. Scores were compared against demographic data using independent sample *t*-tests or ANOVA for categorical variables, and linear regression for continuous variables.

The IBD-KID2 scores of the children with IBD, and their siblings, were explored for associations with their parents’ scores using the Pearson correlation coefficient, with results closer to 1 indicating good correlation, the significance of this result was also derived.

Reliability between the baseline and follow-up completion of IBD-KID2 was measured using paired sample *t*-tests as well as the intraclass correlation coefficient (ICC). Reliability was established if there was no significant difference between mean scores between the 2 administrations of IBD-KID2. Reliability using the ICC score would be moderate if between 0.5 and 0.75, good if between 0.75 and 0.9, and excellent if greater than 0.9 (12).

Generalizability between the 3 countries was first examined by comparing population demographics using categorical and linear variables for each country to establish the degree of similarity between the cohorts using Chi-square tests and ANOVA where applicable. Mean IBD-KID2 scores were then calculated and compared for participant groups in each country and were tested for their association with independent variables between countries using univariate analysis and presented as regression values for linear variables, mean difference for categorical variables, and F value (group variance) for nominal variables, with a high F value indicating rejection of the null hypothesis.

An assessment will be made of the scores and answer patterns for all individual children with IBD, siblings, and their parents to determine whether the online IBD-KID2 was answered by the allocated respondent. The results will be presented as the number of individual pairs with matching scores, as well as a subsequent number of those with matching answer patterns.

Results were considered significant at *P* < 0.05 with a 95% confidence interval. Statistical analysis was performed using SPSS for Windows v25 (13) and GraphPad Prism (14).

## RESULTS

### Participants

A total of 130 children with IBD were included in the study. Of these participants, 118 (91%) had their mother participating, and 55 (42%) their father, with 67% having 1 parent and 33% both parents taking part. There were 37 (28%) children with IBD who had at least 1 sibling participating.

### Demographic Data

The demographic distribution of the children with IBD was provided by all but 8 participants (Table 1).

**TABLE 1.** Demographic Data for Children With IBD

Category	Children with IBD*
Age (y)	13.9 (2.4) [8–18]
Time since diagnosis (y)	3.3 (2.8) [0.1–14]
Age at diagnosis (y)	10.6 (3.5) [1.5–16.7]
Sibling age (y)	14.5 (3.5) [9–21]
Gender	
Male	64 (53)
Female	58 (47)
Diagnosis	
CD	78 (64)
UC	39 (32)
IBDU	5 (4)
Parent has IBD	
No	110 (91)
Yes	11 (9)
Parent in a support group	
No	101 (83)
Yes	21† (17)
Sibling has IBD	
No	37 (97)
Yes	1 (3)
Mother education level	
High school	31 (28)
College	35 (32)
University	31 (29)
Postgraduate	12 (11)
Father education level	
High school	14 (28)
College	15 (29)
University	13 (25)
Postgraduate	9 (18)

Categorical data presented as N (%), linear variables as mean years (SD) [range].

*Data provided by 122.

†Eighteen children had 1 parent belonging to an IBD support group, and 3 had both parents.

CD, Crohn disease; IBD, inflammatory bowel disease; IBDU, inflammatory bowel disease unclassified; UC, ulcerative colitis.

### IBD-KID2 Scores

**FIGURE 1.**
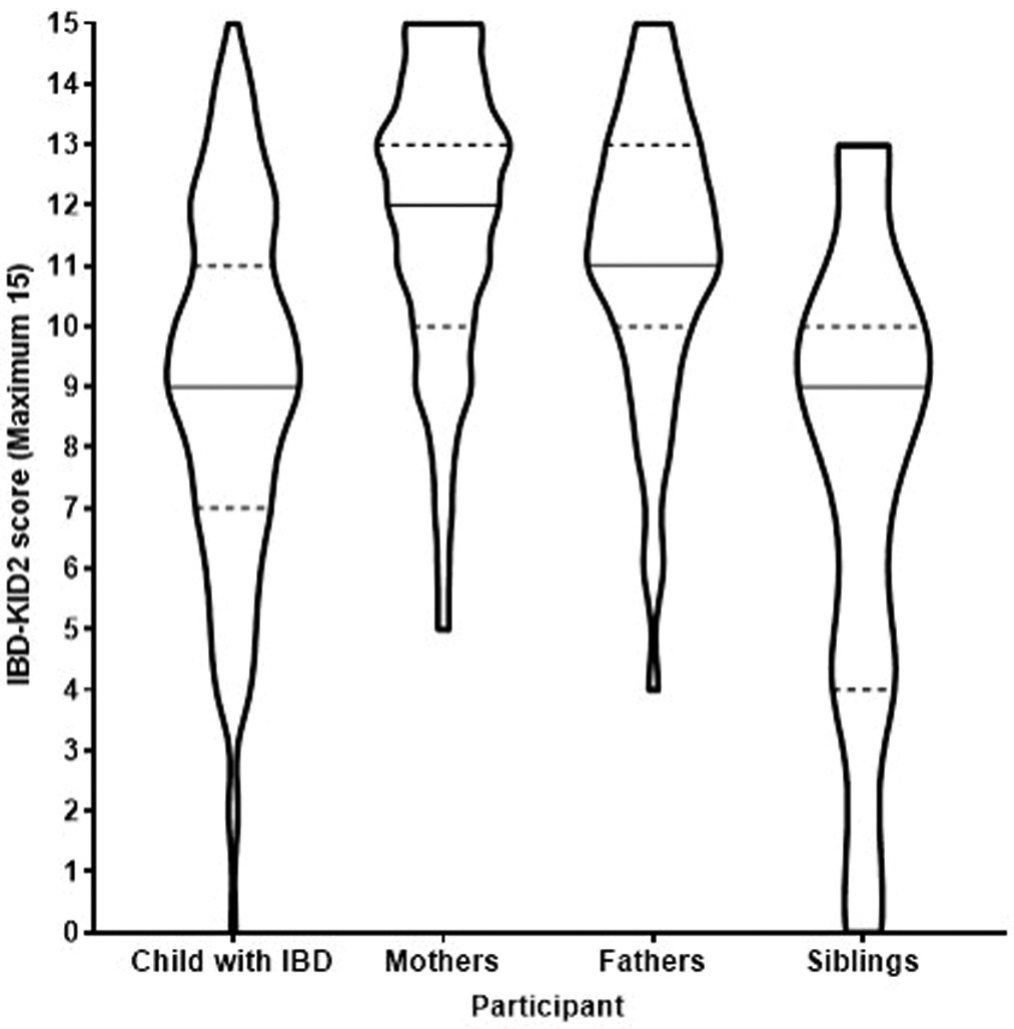
Distribution of IBD-KID2 scores for each participant group. Group shapes represent score distributions, solid line represents median, dotted line 25th and 75th quartile. IBD-KID2, inflammatory bowel disease knowledge assessment tool.

For the overall cohort, the mean IBD-KID2 scores (maximum score 15) for each participant group were calculated, with the hierarchy of scores between family members being (high to low) mothers (mean 11.8, SD 2.4), fathers (mean 11.2, SD 2.3), children with IBD (mean 9.1, SD 2.9), and siblings (mean 7.5, SD 3.8) (Fig. 1). Post-hoc analysis showed children with IBD and their siblings had significantly lower scores than their parents (*P* ≤ 0.005), and children with IBD scored significantly higher than their siblings (*P* = 0.009), but no difference was found between mothers and fathers (*P* = 0.561).

### Independent Variables

The scores achieved on IBD-KID2 by each participant group were examined for their association with a number of independent linear and categorical variables (Table 2). Of note is that the effect of having a sibling with IBD could not be examined as there was only 1 sibling matching this category. The scores of children with IBD and their siblings were associated with age and the scores of children with IBD with their age at diagnosis. The mother’s scores in this cohort were associated with their own education level, and the father’s scores, as well as the children with IBD scores, were associated with the father’s education level.

**TABLE 2.** Effect of Independent Variables on IBD-KID2 Scores for Each Participant Group

Group		
**Linear variables**	***R***	***P***
Participant age		
Child with IBD	0.30	*0.001*
Mother	0.12	0.21
Father	0.04	0.77
Sibling	0.47	*0.002*
Age of child with IBD		
Mother	0.164	0.084
Father	0.135	0.382
Sibling	0.256	0.101
Age of child with IBD at diagnosis		
Child with IBD	0.19	*0.04*
Mother	0.14	0.14
Father	0.23	0.10
Sibling	0.30	0.07
Time since diagnosis		
Child with IBD	0.03	0.75
Mother	0.04	0.71
Father	0.21	0.15
Sibling	0.10	0.52
**Categorical variables**	**MD**	***P***
Gender (M/F)		
Child with IBD	0.1	0.86
Parent	−0.59	0.13
Sibling	−0.81	0.49
Parent has IBD (N/Y)		
Child with IBD	0.79	0.40
Mother	−0.45	0.61
Father	0.15	0.92
Sibling	−2.7	0.19
Parent in a support group (N/Y)		
Child with IBD	0.31	0.66
Mother	−1.1	0.05
Father	−0.92	0.53
Sibling	−0.66	0.68
**Nominal variables**	**F**	***P***
Nominal		
Diagnosis		
Child with IBD	0.75	0.47
Mother	0.79	0.46
Father	0.12	0.89
Sibling	2.63	0.09
Mother education level		
Child with IBD	2.12	0.10
Mother	5.6	*0.001*
Sibling	0.47	0.70
Father education level		
Child with IBD	3.67	*0.02*
Father	2.84	*0.04*
Sibling	0.32	0.81

Italicized values are significant at *P* < 0.05. F = F value, MD = mean difference, *P* = *P* value, *R* = linear regression score.

IBD, inflammatory bowel disease.

Further analysis of parent education levels showed that mean scores for both mothers and fathers with a high school education were significantly lower than those with postsecondary education (Mothers: MD −1.9, *P* = 0.01; Fathers MD −1.7, *P* ≤ 0.005). Similarly, the effect of the father’s education level on the IBD-KID2 score of their child with IBD was significant for fathers with postsecondary education (MD −2.1, *P* = 0.01).

### Score Correlations

The correlation of IBD-KID2 scores between the child participant groups and their parents showed that scores of children with IBD were weakly, but positively, correlated with those of their mother (*R* 0.317, *P* ≤ 0.005) and sibling (*R* 0.345, *P* = 0.03) using matched family pairs. No other participant scores were significantly correlated.

### Test–Retest Reliability

Seventy-four (57%) of the cohort of 130 children with IBD completed a repeat IBD-KID2 assessment. The mean number of days between the completion of the baseline and repeat IBD-KID2 administration was 28 days. The mean IBD-KID2 scores between the baseline and repeat administrations were not significantly different (MD 0.3, *P* = 0.912) with a high ICC score (ICC = 0.851, *P* ≤ 0.005) thus establishing test–retest reliability for the target population of IBD-KID2.

### Generalizability

To determine whether the study cohorts from the 3 countries were comparable participant demographic data were assessed using categorical and linear variables (Supplemental Table 1, Supplemental Digital Content, *http://links.lww.com/PG9/A49*).

**FIGURE 2.**
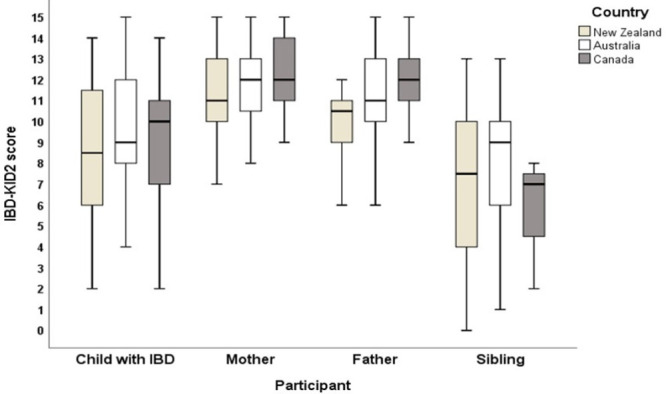
IBD-KID2 scores for participant groups in each country. IBD-KID2, inflammatory bowel disease knowledge assessment tool.

The mean IBD-KID2 scores for each group in each country were calculated and compared using an ANOVA, which showed there were no significant score differences between locations for any participant groups: children with IBD (*P* = 0.423), mothers (*P* = 0.427), fathers (*P* = 0.063), or siblings (*P* = 0.242) (Fig. 2).

The IBD-KID2 score differences for each participant group in the 3 countries were explored for their association with all independent variables, showing no significant differences (Supplemental Table 2, Supplemental Digital Content, *http://links.lww.com/PG9/A49*).

### Patterns of Knowledge

The frequency of correct answers given to each IBD-KID2 item was reviewed between participant groups for the overall cohort. This showed that for each participant group, the number of items answered correctly by at least 50% of each group were: children with IBD 9/15, mothers 15/15, fathers 15/15, and siblings 7/15 (Supplemental Table 3, Supplemental Digital Content, *http://links.lww.com/PG9/A49*).

The frequency of correct IBD-KID2 responses was compared for the children with IBD from each country, which illustrates similar patterns of knowledge between the 3 countries and highlights where consistent knowledge deficiencies exist in this cohort (Supplemental Figure 1, Supplemental Digital Content *http://links.lww.com/PG9/A50*).

### Respondent Completion

A visual inspection of family scores was made that revealed 29 pairs of children and parent scores were the same (23 children with IBD, 6 siblings). For the children with IBD 6 of the 23 pairs had identical answer patterns as 1 parent, and none of the siblings had identical answer patterns.

## DISCUSSION

This external validity study used IBD-KID2 to assess the knowledge levels of a number of family participant groups in 3 geographically diverse countries. We have shown IBD-KID2 to be an appropriate tool for measuring the IBD knowledge levels of children with IBD, as well as their mothers, fathers, and siblings, and has added to the previous literature (11) to establish that IBD-KID2 is a valid, reliable and generalizable tool.

The hierarchy of knowledge levels between participant groups in this study mirror those found in previous studies, as well in other disease groups, whereby parents had significantly higher scores than their children (8–10,15). No studies were identified that had tested knowledge levels of the siblings of children with IBD using a validated assessment tool; however, among children with cystic fibrosis (CF) the same hierarchy was demonstrated as in this study: mothers had the highest scores, followed by fathers, children with CF, then siblings (16). The knowledge levels of the participant groups in this study add to that established in the original validation paper of IBD-KID2 (11), and subsequent research, to show the hierarchy of IBD knowledge to be (highest to lowest): medical staff, mothers, fathers, children with IBD, members of the public (17), siblings of children with IBD, hospital administration staff, and children without IBD. These data may be used as a benchmark in future research using IBD-KID2.

The finding of a significant correlation between the scores of children with IBD and their mothers in this cohort has been shown in a previous study (8). This finding may be positively associated with the effective delivery of education by the health care providers, with mothers and their children with IBD being the more common recipients of this input. This finding is also consistent with the belief of parents that they are the primary source of disease and treatment information for their child (18). The IBD-KID2 scores of the siblings in this study cohort did not correlate with either parent, with possible explanations being that siblings gain knowledge mainly through that provided indirectly through their parents, or through the experience of living with a child with a chronic illness (19–21). This finding is important as the provision of disease and treatment information, as well as good parent-sibling communication about the illness, are protective factors against psychosocial issues among siblings (1,3,6,7). The positive association between sibling knowledge and increasing age in this study suggests that as the siblings grow older, they may independently seek information on the condition. It has been shown that the internet is the primary source of health information for adolescents (22) and they may be using this medium in a desire to better understand their brother’s or sister’s IBD. To maximize sibling knowledge, it has been shown that sibling education programs are effective at increasing knowledge of a condition (19) and a sibling session at organized events such as residential IBD camps should be considered.

Only 24 (15%) of the parents in this study cohort reported that they belonged to an IBD support group, with a significantly higher percentage being in New Zealand compared to Australia and Canada; however, there was no association between this factor and IBD-KID2 scores. In this overall study cohort, the IBD-KID2 scores for mothers belonging to a support group were approaching significance (*P* = 0.054), but this variable had no impact on the IBD-KID2 scores of the child with IBD. This is in contrast with previous research that found children’s scores were significantly higher when their mother belonged to an IBD support group even when the mother’s score showed no association (8). In the adult IBD population, belonging to a support group has shown significant positive effects on knowledge scores (23–26). Previous research has highlighted that attendance at an education program for parents of children with IBD was effective at improving IBD-KID2 scores overall (27). However, 2 items in IBD-KID2 saw no rise in correct answers after the education sessions and further investigation showed these topics were not specifically covered in the program. This highlights that unless support groups are actively providing disease-specific information to parents and children with IBD, there should be no expectation of greater IBD scores. Rather, that support groups may be aimed at improving family well-being, and that disease-specific knowledge should be provided by the clinical team and considered an ongoing, tailored endeavor to benefit each individual family.

### Limitations

The primary limitation of this study was that the countries selected for participation represent English-speaking developed countries, which therefore limits generalizability to areas not matching these criteria. Future research aims to address this limitation by translating IBD-KID2 into languages commonly used throughout the world, thereby maximizing the centers that can incorporate knowledge assessments into their clinical IBD care and research activities.

The use of IBD-KID2 to assess parent knowledge could be considered to skew their scores towards the upper limits due to it being a child-orientated assessment tool. However, adult IBD knowledge assessment tools such as the Crohn and Colitis Knowledge Score (CCKNOW) and the Knowledge Questionnaire contain items that would only be relevant to adults or older adolescents with IBD and parents may not have been exposed to this information yet (23,26). Previous research has shown that parents of children with IBD score approximately 60% on the CCKNOW (10), and in this study cohort their IBD-KID2 scores were higher at 79% for mothers, and 75% for fathers, suggesting that while the IBD-KID2 questions may be easier, they may also be more relevant to them due to the age of their children with IBD (8 years and up in this cohort).

Another potential confounder in this study is the potential for parents to have completed their children’s online IBD-KID2 assessment, or provided assistance, with the aim of artificially inflating their scores. For those children with the same score as a parent but dissimilar patterns, it may be surmised that these were completed by the correct participant. For those 6 with identical answer patterns, it is tempting to speculate that these were completed by the same person, but these pairs represent a small proportion (4.6%) of the cohort of children with IBD.

### Strengths

Implementing IBD-KID2 at multiple sites across 3 geographically diverse countries has provided data to establish external validity. No significant difference in scores was found between the counties for any participant group, adding confidence to the use of IBD-KID2 in the wider population of children with IBD. Lack of demonstrable influence of most independent variables adds to the confidence of generalizability, it is not impaired for those most recently diagnosed, and not related to diagnosis, demonstrating that individual items do not skew to those with a particular IBD subtype.

### Conclusion

This is the first study to establish IBD knowledge levels of the family unit using a standardized tool (IBD-KID2) and to identify independent variables that influence their scores. We have highlighted areas where family knowledge is compromised, in particular siblings of children with IBD. As improved disease-specific knowledge in siblings has been demonstrated to improve their emotional and behavioral outcomes further studies on including siblings in discussions regarding IBD, and their attendance at residential camps for children with IBD, is needed (28,29). When disseminated, this research may enable pediatric gastroenterologists to apply the findings with confidence to their local setting and to begin using IBD-KID2 as a clinical and research knowledge assessment tool.

## Supplementary Material


