# Supplementary material for: Assessment of Disease-related Knowledge Among Children With Inflammatory Bowel Disease and their Family Using IBD-KID2: Evaluating Tool Generalizability
Source: JPGN Rep. 2021 Jul 12;2(3):e093. doi: 10.1097/PG9.0000000000000093 (PMC10191511; doi:10.1097/PG9.0000000000000093)
Supplement: Supplementary file 1 [file pg9-2-e093-s001.pdf]

Appendix 1 - Supplementary Digital Content. IBD-KID2.
© 2020 Vernon-Roberts. All rights reserved.

The
Inflammatory **B**owel **D**isease **K**nowledge **I**nventory **D**evice Version **2** (IBD – KID2)

Instructions:

★ This is not a test so don’t worry about getting the answers wrong.

★ Please put a circle around the letter of one answer. If you don’t know the answer put a circle around ‘don’t know’.

★ Please answer the questions by yourself. If you need someone to read the questions to you that is ok, but we need your answers.

★ Please don’t study before answering the questions or ask for help to get the right answer.

★ Take as long as you like to finish IBD-KID2, but please finish the questions all at the same time. Don’t leave half for later or another day.

★ Thank you for taking the time to fill this in.

1) From start to finish, the correct order of the gut is.:
   a) Mouth → stomach → oesophagus → large bowel → small bowel → anus

b) Mouth → oesophagus → stomach → large bowel → small bowel → anus
c) Mouth → oesophagus → stomach → small bowel → large bowel → anus
d) Don’t know

2) Doctors and scientists know what causes IBD.
   a) True
   b) False
   c) Don’t know

3) Stress can trigger an IBD flare.
   a) True
   b) False
   c) Don’t know

4) The reason you might have a colonoscopy of your large bowel is to:
   a) Look for disease
   b) Remove part of it
   c) Apply drugs inside it
   d) Don’t know

5) IBD can affect other organs, not just the gut.
   a) True
   b) False
   c) Don’t know

6) Which <u>one</u> fact about Osteoporosis (weak bones) is true?
   a) It doesn’t affect males or young women
   b) If I drink plenty of milk I won’t get it
   c) It can be caused by IBD
   d) Don’t know

7) IBD that is in remission can slow down a young person’s growth.
   a) True
   b) False
   c) Don’t know

8) How do biologic drugs work?
   a) They reduce the chance of infections
   b) They block the chemicals or cells that cause inflammation
   c) They help the body absorb enough nutrients
   d) Don’t know

9) If a person with IBD has had no symptoms for a few months they should stop taking their drugs.
   a) True
   b) False

c) Don’t know

10) If both parents have IBD their children will develop IBD.
   a) True
   b) False
   c) Don’t know

11) Which <u>one</u> fact about complementary and alternative products is true? *(example – herbal drugs).*
   a) They may interact with prescribed drugs.
   b) They are natural so do not have side effects.
   c) They are all safe to use with prescribed drugs.
   d) Don’t know

12) If you get side effects from taking steroids you should stop taking them at once.
   a) True
   b) False
   c) Don’t know

13) Not eating some foods will stop your IBD getting worse. (*example - milk*).
   a) True
   b) False
   c) Don’t know

14) Which <u>one</u> fact about IBD surgery is true?
   a) All people with IBD will need surgery
   b) Surgery is not helpful for people with IBD
   c) Surgery is helpful for some people with IBD
   d) Don’t know

15) People with IBD can absorb all the nutrients they need if they eat the right foods.
   a) True
   b) False
   c) Don’t know

Thank you for answering our questions! ☺

Table 1, Supplementary Digital Content: Demographic variables of the children with IBD in each country.

Categorical variables presented as N (%), linear variables as mean years (SD).
* Some children had more than one parent in an IBD support group.

	Category	New Zealand N=28 (22%)	Australia N=57 (44%)	Canada N=45 (34%)	P value
Gender	Male	15 (54)	26 (51)	23 (54)	0.31
	Female	13 (46)	25 (49)	20 (46)	
Diagnosis	CD	20 (71)	29 (57)	29 (68)	0.10
	UC	8 (29)	17 (33)	14 (32)	
	IBDU	0	5 (10)	0	
Parent has IBD	Yes	3 (11)	7 (14)	1 (3)	0.29
	No	25 (89)	43(86)	30 (97)	
Parent in a support group*	Yes	9 (29)	10 (14)	5 (10)	*0.03*
	No	33 (79)	60 (86)	46 (90)	
Mother education level	High school	10 (36)	15 (32)	2 (9)	0.31
	College	6 (21)	15 (32)	10 (46)	
	University	8 (29)	13 (28)	6 (27)	
	Postgraduate	4 (14)	4 (9)	4 (18)	
Father education level	High school	6 (43)	6 (26)	2 (14)	0.58
	College	3 (11)	6 (26)	6 (43)	
	University	4 (14)	6 (26)	3 (21)	
	Postgraduate	1 (4)	5 (22)	3 (21)	
	Child with IBD age	13.7 (2.0)	13.9 (2.4)	13.4 (2.8)	0.64
	Mother age	45.9 (5.1)	47.6 (7.0)	45.7 (5.8)	0.35
	Father age	48.2 (4.5)	50.0 (7.3)	46.9 (6.3)	0.34
	Sibling age	15.2 (3.6)	14.1 (3.5)	12.2 (1.0)	0.40
	Time since diagnosis	2.8 (2.9)	3.7 (3.3)	3.0 (2.3)	0.31
	Age at diagnosis	10.9 (3.5)	10.2 (3.7)	10.4 (3.6)	0.69

Table 2, Supplementary Digital Content. Significance (p value) of the association between IBD-KID2 scores and independent variables between participant groups and country.

* Insufficient numbers for comparisons

**Variable**	**Child with IBD**	**Mother**	**Father**	**Sibling**
**Diagnosis**	0.82	0.47	0.26	0.76
**Gender**	0.57	0.41		0.97
**Age**	0.43	0.49	0.08	0.16
**Age diagnosis**	0.30	0.51	0.05	0.20
**Time diagnosis**	0.43	0.49	0.09	0.20
**Parent has IBD**	0.17	0.85	*	0.70
**Parent in a support group**	0.51	0.98	*	0.24
**Mother education**	0.59	0.69	-	0.07
**Father education**	0.86	-	0.98	0.36

Table 3. Supplementary Digital Content. Percentage of correct answers given to IBD-KID2 for each participant group.

EIM: Extra-intestinal manifestations

CAM: Complementary and alternative medicines

Item	Child with IBD % score	Mother % score	Father % score	Sibling % score
1: Gut order	59	75	62	44
2: IBD cause	60	86	84	54
3: Stress	62	87	84	54
4: Colonoscopy	92	98	98	72
5: EIM	62	69	64	51
6: Osteoporosis	58	70	67	56
7: Growth	47	60	55	40
8: Biologics	46	75	58	37
9: Stopping drugs	90	98	98	84
10: Parents	69	81	91	44
11: CAM	47	80	76	42
12: Steroids	48	70	78	40
13: Food triggers	45	67	60	37
14: Surgery	88	98	91	70
15: Nutrients	35	61	51	30
