# Supplementary figures and images for: Assessment of Disease-related Knowledge Among Children With Inflammatory Bowel Disease and their Family Using IBD-KID2: Evaluating Tool Generalizability
Source: JPGN Rep. 2021 Jul 12;2(3):e093. doi: 10.1097/PG9.0000000000000093 (PMC10191511; doi:10.1097/PG9.0000000000000093)

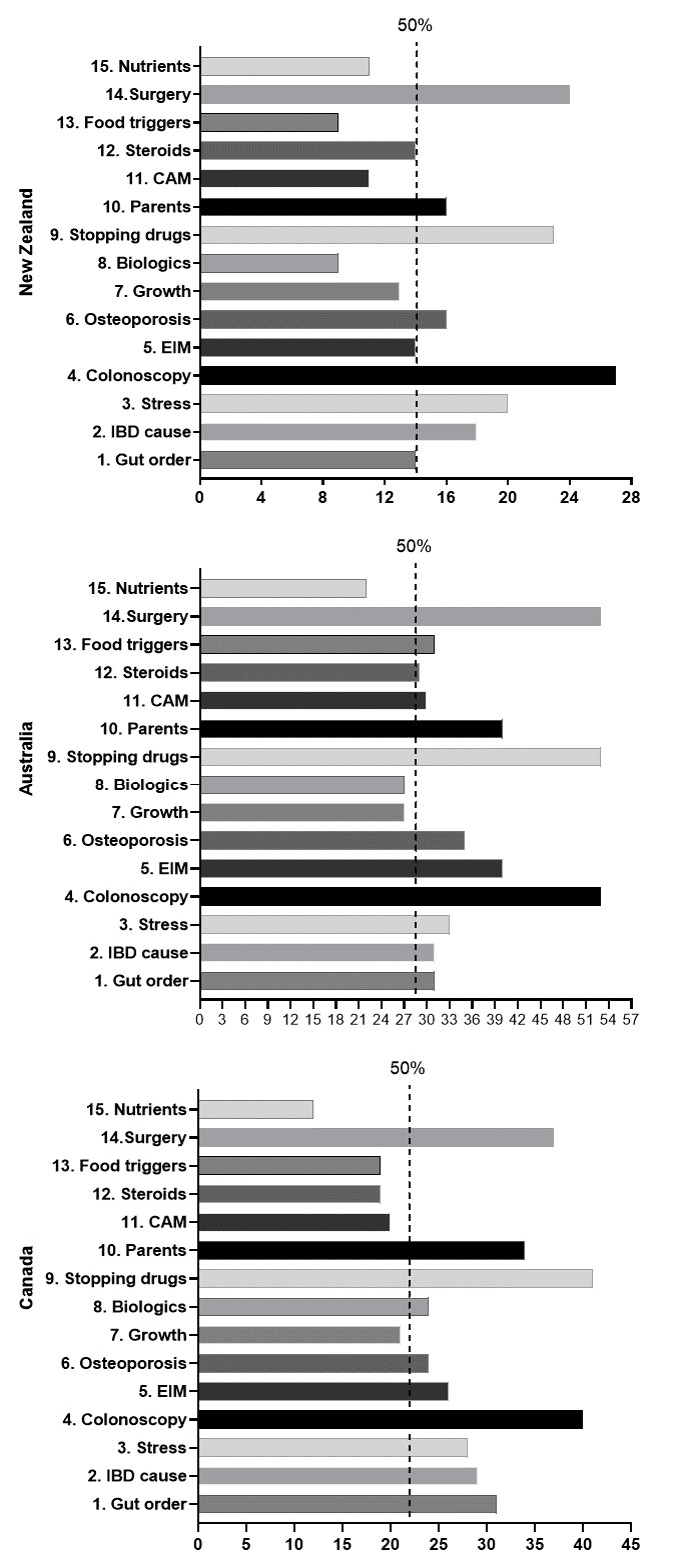

Supplement: Supplementary file 2 [file pg9-2-e093-s002.jpg]
